# Role of Trachway versus Conventional Modes of Intubation in Difficult Airway Management in COVID-19 Setups

**DOI:** 10.1155/2021/6614523

**Published:** 2021-02-25

**Authors:** Meng-Yu Wu, Giou-Teng Yiang, Jian-Yu Ke, Chien-Sheng Chen, Po-Chen Lin, Yu-Long Chen

**Affiliations:** ^1^Department of Emergency Medicine, Taipei Tzu Chi Hospital, Buddhist Tzu Chi Medical Foundation, 231 New Taipei, Taiwan; ^2^Department of Emergency Medicine, School of Medicine, Tzu Chi University, 970 Hualien, Taiwan; ^3^Department of Emergency and Critical Care Medicine, Fu Jen Catholic University Hospital, Fu Jen Catholic University, New Taipei City, Taiwan; ^4^School of Medicine, College of Medicine, Fu Jen Catholic University, New Taipei City, Taiwan

## Abstract

Difficult airway management in critically ill patients remains a difficult task associated with high morbidity and mortality rates. In difficult airway populations, prompt effective intubation is more important to prevent hypoxia and neurological injury. During the ongoing COVID-19 pandemic, prolonged intubation time and repeated intubation can lead to an increase in the risk of infection. Therefore, digital devices can shorten intubation times and decrease the risk of infection among clinical staff. The advantages of the Trachway videolight intubating stylet suit these conditions. Trachway stylet intubation is an effective method for video laryngoscopy to enhance patient safety and improve the intubation success rate. However, a few studies have focused on the effect of stylet intubation by reducing repeated intubation and oxygen desaturation. In this study, we reviewed current data of Trachway intubation and shared our four major training scenarios in Taipei Tzu Chi Hospital via the Trachway videolight intubating stylet system for emergency intubation, comparing them with other modes of intubation.

## 1. Introduction

During this critical era of the ongoing COVID-19 pandemic, airway intubation faces a challenge for high risk of COVID-19 infection, increased number of infected patients, straining health-care systems, and crashing local economies. Several types of video laryngoscopy have been reported to shorten intubation times and decrease the risk of infection among clinical staff [1–3]. The Trachway videolight intubating stylet system (Biotronic Instrument Enterprise Ltd., Tai-Chung, Taiwan) is a J-shape stylet fabricated using stainless steel, which is promoted for tracheal intubation because it easily passes through the oral cavity. The Trachway consists of three parts: a video recorder at the end of the stylet, grip, and screen. The video stylet can allow physicians to visualize the vocal cords and endotracheal tube passage. In general intubation, the patient is placed in a sniffing position for conventional direct laryngoscope intubation. However, direct laryngoscope intubation is difficult under several conditions (cervical spinal cord injuries, facial injury, and temporomandibular joint rigidity). The advantage of the Trachway videolight intubating stylet suits these conditions and provides effective intubation, compared to direct laryngoscopy. In addition, the Trachway videolight intubating stylet can also be transformed to video laryngoscopy by changing the J-shape stylet to the laryngoscope blade. Thus, one system is made to have two effective intubation methods. The transformation from Trachway video laryngoscopy to Trachway video intubating stylet is easy and quick. In this ongoing pandemic, the Trachway may significantly decrease the risk of COVID-19 infection by improving the preparedness of frontline doctors during emergency intubation, especially in emergency medical students, residents, and fellows. In this study, we reviewed current data of Trachway intubation and shared our data in Taipei Tzu Chi Hospital. We equally used the Trachway videolight intubating stylet system to create four major scenarios for emergency intubation in COVID-19 patients, comparing them with other modes of intubation.

## 2. Methods

Our training program was held from August 2019 to June 2020. It included four training courses (Table 1). The manikin was placed on a standard stretcher, and our program was carried out in the emergency department. The four training courses were carried out on Laerdal Airway Management Trainer™ (Laerdal Medical Ltd, Orpington, UK) for medical students, residents, and fellows. In difficult airway management, the manikin was set up with an enlarged manikin tongue to create an upper airway obstruction. The four training courses included direct laryngoscopy, video laryngoscopy, Trachway stylet, and surgical airway. Before the four scenarios, all trainees participated in two-hour training lectures. After training lectures, the scenarios were performed immediately. In each scenario, we requested all trainees to perform endotracheal intubation thrice. In the first scenario, we compared the efficacy of the Trachway stylet to direct laryngoscopy and video laryngoscopy for intubation in normal adults. In the second scenario, we compared the Trachway stylet to direct laryngoscopy, video laryngoscopy, and surgical airway for difficult airway management. In the third scenario, we used the Trachway stylet for nasal intubation in a difficult airway. In the fourth scenario, we simulated the intubation in COVID-19 patients by direct laryngoscopy and video laryngoscopy in protective cover (Table 1).

## 3. Result

### 3.1. Trachway System in Orotracheal Intubation

**Figure 1:**
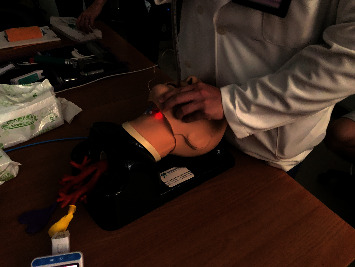
The tip of the Trachway intubating stylet is also equipped with the light, just like a lightwand, for guiding into the trachea by transillumination of the neck tissues.

The Trachway intubating stylet for digital intubation was induced by Ong et al. [4] in 2009, and they reported that the duration of intubation is approximately 21–25 seconds in manikins, which is adequate for clinical use. The Trachway intubating stylet provided an alternative tool for intubation. The Trachway intubating stylet is an intubating stylet just like a lightwand but with a camera for direct observation (Figure 1). During intubation, the Trachway stylet is inserted along the midline of the tongue to view the glottis. After the stylet passed through the glottis, the vocal cords would come into view, and then, the endotracheal tube is slid into the trachea. Using Trachway stylet intubation may not need to change head or neck position to facilitate intubation, and in a population with limited mouth opening, it may provide more easy intubation.

### 3.2. Trachway System in Nasotracheal Intubation for Difficult Airway

**Figure 2:**
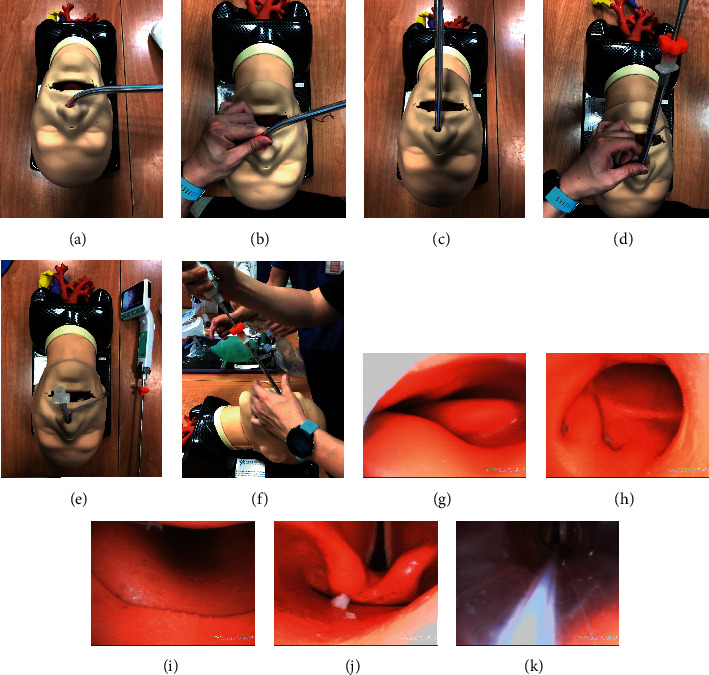
Using a Trachway stylet for nasointubation. (a) Inserting the tip of the endotracheal tube with a Trachway stylet from the selected nostril, (b) using the left hand to control the force and prevent from epistaxis, (c) 90-degree rotation and elevation of the Trachway stylet at the same time to midline, (d) making the curve of the Trachway stylet fit the anatomy curve of the nasopharynx and oropharynx, (e, f) the tip of the endotracheal tube was advanced to the target depth in a routine manner, (g, h) the view of inserting the tip of the endotracheal tube with the Trachway stylet in the nasopharynx, (i) the view of the tip of the endotracheal tube in the oropharynx, (j) the view of the tip in the laryngopharynx, and (k) the view of the tip of the endotracheal tube passing through the vocal cord.

**Figure 3:**
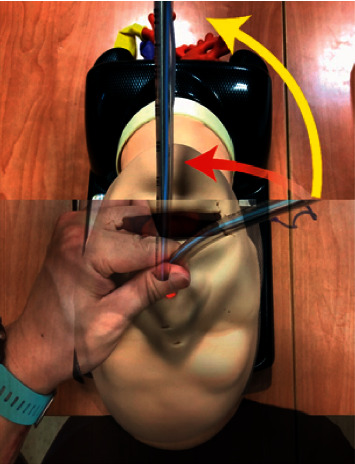
After the tip of the endotracheal tube is inserted into the selected nostril, 90-degree rotation (red arrow) and elevation (yellow arrow) of the Trachway stylet at the same time to midline make the curve to fit the anatomy curve of the nasopharynx and oropharynx.

In recent years, the efficacy of the Trachway stylet system in nasotracheal intubation has been investigated. Hsu et al. [5] reported that 100 patients for oromaxillofacial surgery received nasotracheal intubation (NCT01917409) in comparison with the Macintosh laryngoscope and Trachway stylet assistance. In the Trachway stylet group, the total intubation time and duration of using tools to advancing the tube into the trachea were significantly shorter, compared to the laryngoscope group. The median score of the modified nasotracheal intubation difficulty scale was higher in the laryngoscope group. During intubation, up to 38% of patients needed cuff inflation and 54% patients needed BURP maneuver in the laryngoscope group, but no patients needed assistance in the Trachway group. The mean total intubation time in the Macintosh laryngoscope is about 2 min for trainees and 1 min for experienced intubators with Magill forceps, and the Trachway stylet nasointubation has a shorter mean total intubation time, 32.3 sec [6, 7]. Our study then provides supportive evidence for Trachway stylet nasointubation in difficult airway management, especially in patients with limited mouth opening. In our third scenario, we used the Trachway stylet for nasal intubation in a difficult airway (Figure 2). First, secretions of the oropharynx or hypopharynx may impede the view of the trachea. Suction with the DuCanto catheter, at least using Yankauer, may improve the view. Second, slowly inserting the tip of the endotracheal tube with the Trachway stylet from the selected nostril by the left hand is important to prevent epistaxis, which may cause poor visualization of the pharynx. The tip of the Trachway stylet is recommended to hind the inner tip of the endotracheal tube to prevent blurring. Finally, the Trachway is often held in the right hand, and the left hand can assist the insertion of the tip of the endotracheal tube through the left nostril. After the tip of the endotracheal tube passes through the nostril, it rotates 90° and elevates while crossing the midline as it fits the anatomy curve of the nasopharynx and oropharynx (Figure 3). In view of the laryngopharynx, the tip of the endotracheal tube was advanced to the target depth in a routine manner.

### 3.3. Trachway System in COVID-19 Intubation

**Figure 4:**
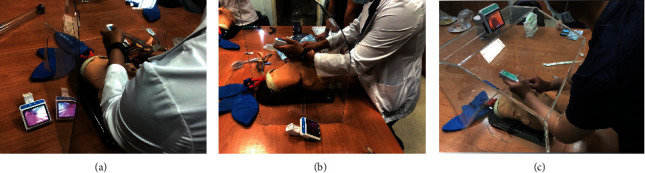
(a) Trachway with a stylet or video laryngoscope is more effective to prevent contamination wirelessly. (b, c) Trachway system intubation is suitable to different acrylic protective covers.

In the fourth scenario, we simulated the intubation in COVID-19 patients. The trainers were intubated in an acrylic protective cover designed by Dr. Lai Hsien-yung (available at https://sites.google.com/view/aerosolbox/home) to decrease the risk of contamination [8]. This device consists of an acrylic box with an opening side to receive the patient's head and neck and two small holes for the intubator's hands on the opposite side. The monitor of the Trachway can be disconnected to the stylet and placed out of the box to prevent risk of contamination. A Trachway with a stylet or video laryngoscope was more effective for intubation (Figure 4). In the acrylic box, direct laryngoscopy is more difficult for intubation due to limitations of hand motion and visualization in small spaces. Video laryngoscopy is more suitable for the modified acrylic protective cover. To prevent risk of contamination, the Trachway system could wirelessly connect with the monitor. In addition, this system can connect with more than one monitor at the same time, thereby providing an effective teaching tool for medical students.

In this study, we also searched the PubMed databases for articles published from inception to October 2020. No limits were applied to our Boolean search strategy using the keywords in bracket (“Trachway,” “Trachway intubation,” ”difficult airway,” “video laryngoscopes,” and “intubation”). References from retrieved articles were also examined to identify other relevant articles. Studies were included if they used Trachway intubation systems. Studies were excluded if they were irrelevant to the study's aim or were animal studies. In total, 23 articles were included in the discussion analysis.

## 4. Discussion

Airway management in critically ill patients, suspected to be infected with COVID-19, by emergency physicians is a challenge, especially in difficult airways. In the current concept, shortening the intubation time and indirect intubation would decrease the risk of contamination. The role of video laryngoscopy and digital video stylet has become increasingly important for orotracheal intubation in high-risk patients [1–3]. Trachway stylet intubation is friendly for inexperienced physicians, and the effect of Trachway stylet intubation is similar to that of other video laryngoscopes. In Tseng et al.'s [9] manikin study, 36 medical students without previous experience in tracheal intubation were included to compare the Trachway intubation stylet and airway scope video laryngoscope. Overall success rates did not differ significantly between Trachway stylet intubation and video laryngoscopy. In intubation time, there was no significant difference as well. Kim et al. [10] found that the success rate for tracheal intubation is similar, but stylet intubation provided faster and easier intubations than the airway scope video laryngoscope. On comparing the Trachway intubating stylet and Macintosh direct laryngoscopy in a manikin study with 38 nurse anesthesiologists, it was revealed that the Trachway intubating stylet had shorter intubation times and proved easier with intubations than direct laryngoscopy in difficult airway management, but no difference was observed in the normal airway. In addition, the Trachway intubating stylet did not have any complication event as the direct laryngoscope [11]. In Cooney et al.'s [12] study, attending and resident emergency physicians were included for intubation of a difficult airway in high-fidelity simulated patients with stylet intubation in comparison with direct laryngoscopy. The results showed a 100% first attempt success rate and a lower cumulative attempt time in the stylet intubation group. In current data, a Trachway with Macintosh laryngoscopy for intubation is recommended for medical learners. However, in experienced intubators (residents and fellows), the learning curve of Trachway stylet intubation is shorter. Hence, Trachway stylet intubation is a more effective, faster, and easier method, especially for difficult airway management.

In difficult airway management, awake intubation is a good choice but requires a skilled operator to perform it. Many video intubation devices provide an alternative method for physicians to awake tracheal intubation. The Trachway video stylet has a similar size but more rigidity compared to fibers. During Trachway awake intubation, the traditional transtracheal block, spray-as-you-go technique, is not attempted because of the lack of a working channel in this device. A novel modified method for Trachway awake intubation is needed [13]. The Trachway stylet and a 6 Fr suction tube were inserted into the lumina of a size 7 or larger endotracheal tube through the side-arm orifice of the double-swivel connector. After administration of lidocaine injection into the trachea from inner suction tube, an endotracheal tube was further advanced into the trachea. The Trachway stylet and suction tube were removed without the endotracheal tube. Poor visualization is a major problem in Trachway awake intubation. Oral secretions during the intubation process may cause poor visualization even after oral secretion. In obese or limited mouth opening population, excess airway tissue also leads to this problem. A jaw thrust or head-tilt maneuver may improve visualization during intubation. Poor visualization may prolong duration of intubation and increase discomfort to the conscious patient, especially in obese patients. In facilitating tracheal intubation, the Trachway is reported effective in anesthetized patients with difficult airway. In Hung et al.'s [13] report, we found that Trachway video stylet awake intubation is an alternative tool for difficult airway, especially in an emergency condition [14].

The efficacy of the Trachway stylet system for emergency nasotracheal intubation has been investigated. In a study by Lee et al. [15], 80 patients with limited mouth opening undergoing oromaxillofacial surgery were included and the results showed the mean total intubation time was significantly shorter in the Trachway group. In the modified nasal intubation difficulty scale analysis, only 55% of patients in the fiberoptic intubation group were categorized as having no difficulty with intubation unlike 100% of patients in the Trachway group. There were no significant differences in complication rates (bleeding from the nostril, accumulation of blood in the oropharyngeal space, postoperative sore throat, hoarseness, and pain on swallowing) between the two groups. Trachway stylet nasotracheal intubation provided shorter intubation time, better intubation conditions, and similar complication rates, compared with fiberoptic intubation. The Trachway stylet-assisted nasotracheal intubation was prevented from blind advancement by direct visualization of the path through the nasal cavity. The distal tip of the Trachway stylet can be positioned in the nasotracheal tube to prevent damage to the nasal tissues. Therefore, using the Trachway stylet for nasointubation is an alternative tool for difficult airway management and a rescue method for emergency conditions [16].

## 5. Conclusions

In this study, we shared with our experience and concluded from previous reported studies that the Trachway stylet intubation system provided more effective, faster, and higher success rates of intubation than direct laryngoscopy. In addition, the advantages of the Trachway system included wireless connection, reducing the risk of contamination during airway management in COVID-19 patients.

## Figures and Tables

**Table 1:** Four scenarios of the training program and simulation setting.

Scenarios	Classification	Devices
Scenario 1	Normal intubation	Trachway stylet vs. direct/video laryngoscopy
Scenario 2	Difficult intubation	Trachway stylet vs. direct/video laryngoscopy and surgical airway
Scenario 3	Difficult intubation (nasal intubation)	Trachway stylet
Scenario 4	COVID-19 intubation in protective cover	Direct laryngoscopy vs. video laryngoscopy

## Data Availability

No data were used to support this study.
